# An Introduction to Practical Chemistry, Including Analysis

**Published:** 1849-11

**Authors:** 


### ARTICLE III.

*An Introduction to Practical Chemistry, including Analysis.*—By JOHN E. BOWMAN, Demonstrator of Chemistry in King's College, London. Philadelphia: Lea & Blanchard. 1849. pp. 303., 12 mo. (From the Publishers. For sale by S. C. Griggs & Co.)

For the student of Practical Chemistry we know of no better volume than this. The principles of Chemistry are abundantly treated of in the standard works on the science; but there has been a want of books treating of the mysteries of the laboratory. Chemistry, like other Natural Sciences, is a dull study without demonstration. To have an exact and thorough knowledge of the science, the student must have a practical knowledge of the manipulations of the laboratory. This sort of information is as important to the chemist, as is dissection to the anatomist; and books like the one before us have the same relation to the standard treatises on Chemistry that the *Dissectors* bear to the more elaborate works on Anatomy.— Faraday's *Chemical Manipulations*, which has heretofore been the standard work on Practical Chemistry, is too large; being calculated rather for the teacher than the student; premising as it does, the possesion of extensive and costly apparatus. In this treatise, Mr. Bowman has presented in a clear and concise manner, a description of the Chemical Manipulations necessary to render the student an expert experimenter. There is also sufficient directions for Practical Analysis to render the work of value and interest to the profession at large. M.

